# A type I-F CRISPRi system unveils the novel role of CzcR in modulating multidrug resistance of *Pseudomonas aeruginosa*

**DOI:** 10.1128/spectrum.01123-23

**Published:** 2023-08-30

**Authors:** Shuzhen Chen, Huiluo Cao, Zirui Xu, Jiahui Huang, Zhiqing Liu, Ting Li, Cheng Duan, Weiyan Wu, Yongqi Wen, Lian-Hui Zhang, Zeling Xu

**Affiliations:** 1 Guangdong Province Key Laboratory of Microbial Signals and Disease Control, Integrative Microbiology Research Centre, South China Agricultural University, Guangzhou, China; 2 Department of Microbiology, Li Ka Shing Faculty of Medicine, The University of Hong Kong, Hong Kong, China; 3 Guangdong Laboratory for Lingnan Modern Agriculture, South China Agricultural University, Guangzhou, China; South China Sea Institute of Oceanology Chinese Academy of Sciences, Guangzhou, Guangdong, China

**Keywords:** *P. aeruginosa*, type I-F CRISPRi, efflux pump, CzcR, antibiotic resistance

## Abstract

*Pseudomonas aeruginosa* has abundant signaling systems that exquisitely control its antibiotic resistance in response to different environmental cues. Understanding the regulation of antibiotic resistance will provide important implications for precise antimicrobial interventions. However, efficient genetic tools for functional gene characterizations are sometimes not available, particularly, in clinically isolated strains. Here, we established a type I-F CRISPRi (CSYi) system for programmable gene silencing. By incorporating anti-CRISPR proteins, this system was even applicable to bacterial hosts encoding a native type I-F CRISPR-Cas system. With the newly developed gene-silencing system, we revealed that the response regulator CzcR from the zinc (Zn^2+^)-responsive two-component system CzcS/CzcR is a repressor of efflux pumps MexAB-OprM and MexGHI-OpmD, which inhibits the expression of both operons by directly interacting with their promoters. Repression of MexAB-OprM consequently increases the susceptibility of *P. aeruginosa* to multiple antibiotics such as levofloxacin and amikacin. Together, this study provided a simple approach to study gene functions, which enabled us to unveil the novel role of CzcR in modulating efflux pump genes and multidrug resistance in *P. aeruginosa*.

**IMPORTANCE:**

*P. aeruginosa* is a ubiquitous opportunistic pathogen frequently causing chronic infections. In addition to being an important model organism for antibiotic-resistant research, this species is also important for understanding and exploiting CRISPR-Cas systems. In this study, we established a gene-silencing system based on the most abundant type I-F CRISPR-Cas system in this species, which can be readily employed to achieve targeted gene repression in multiple bacterial species. Using this gene-silencing system, the physiological role of Zn^2+^ and its responsive regulator CzcR in modulating multidrug resistance was unveiled with great convenience. This study not only displayed a new framework to expand the abundant CRISPR-Cas and anti-CRISPR systems for functional gene characterizations but also provided new insights into the regulation of multidrug resistance in *P. aeruginosa* and important clues for precise anti-pseudomonal therapies.

## INTRODUCTION

*Pseudomonas aeruginosa* is an opportunistic human pathogen that can produce many virulence factors such as pyocyanin, elastases, proteases, rhamnolipids, siderophores, and exoenzymes to cause acute and chronic infections frequently in immunocompromised patients (1). It is notorious for the extraordinary capacity of resistance to different antibiotics owing that the pathogen is equipped with a variety of resistance determinants, such as drug efflux pumps, antibiotic-inactivating enzymes, and the ability to grow as biofilms and reduce the uptake of antibiotics (2). It is well-acknowledged that the antibiotic resistance of *P. aeruginosa* is controlled tightly by its abundant signaling systems. Among them, the two-component system (TCS) is an important one to regulate antibiotic resistance in response to diverse environmental cues including nutrient availabilities and host factors (3). For example, the TCS CzcS/CzcR was found as a repressor of OprD, a porin for the entry of carbapenem antibiotics, and is responsible for the increased resistance of *P. aeruginosa* to carbapenems (4, 5).

In addition to the great ability to cause serious infections and develop antibiotic resistance, *P. aeruginosa* is also a bacterial species with recalcitrance to genetic manipulations owing to the deficient homologous recombination capacity particularly in clinically isolated antibiotic-resistant strains with complicated genetic background (6). The CRISPR/CRISPR-associated gene (Cas) systems, which are adaptive immune systems ubiquitously found in prokaryotes, have attracted extensive attention to be repurposed for programmable gene silencing (also known as CRISPR interference, CRISPRi) in many organisms in the past decade (7, 8). CRISPRi has emerged as an exciting tool to rapidly characterize gene functions by repressing target genes at the transcriptional level without manipulating genome sequences. Recently, we developed a chromosomal integrative type I-F CRISPRi system, which enables targeted and efficient gene repression (9). However, owing to the dependence of the specific chromosomal integration site *attB*, the chromosomal integrative type I-F CRISPRi system only works in *P. aeruginosa*. Moreover, a large percentage (>30%) of *P. aeruginosa* strains were predicted to encode type I-F CRISPR-Cas systems in their genomes (10), which inevitably inactivates the existing chromosomal integrative type I-F CRISPRi system in these strains. Hence, a more adaptable type I-F CRISPRi system is desired to achieve gene silencing in different bacterial hosts even strains encoding type I-F CRISPR-Cas systems.

In this study, we aimed to develop a new gene-silencing system based on the type I-F CRISPR-Cas system and expected to use the gene-silencing system for antibiotic resistance research. We firstly explored whether CzcS/CzcR potentially regulates the susceptibility of *P. aeruginosa* to other antibiotics in addition to carbapenems and interestingly discovered a novel function of CzcR in reducing *P. aeruginosa* resistance to the fluoroquinolone antibiotic levofloxacin. To facilitate dissecting the regulatory mechanisms, we then developed a plasmid-based type I-F CRISPRi (CSYi) system that can be readily utilized for targeted gene silencing in multiple bacterial species as well as strains encoding native type I-F CRISPR-Cas system. With the CSYi system, we elucidated the novel role of CzcR in regulating efflux pumps and multidrug resistance in *P. aeruginosa*.

## RESULTS

### CzcR inhibits levofloxacin resistance and the expression of *mexAB-oprM* and *mexGHI-opmD* in *P. aeruginosa*

**Fig 1:**
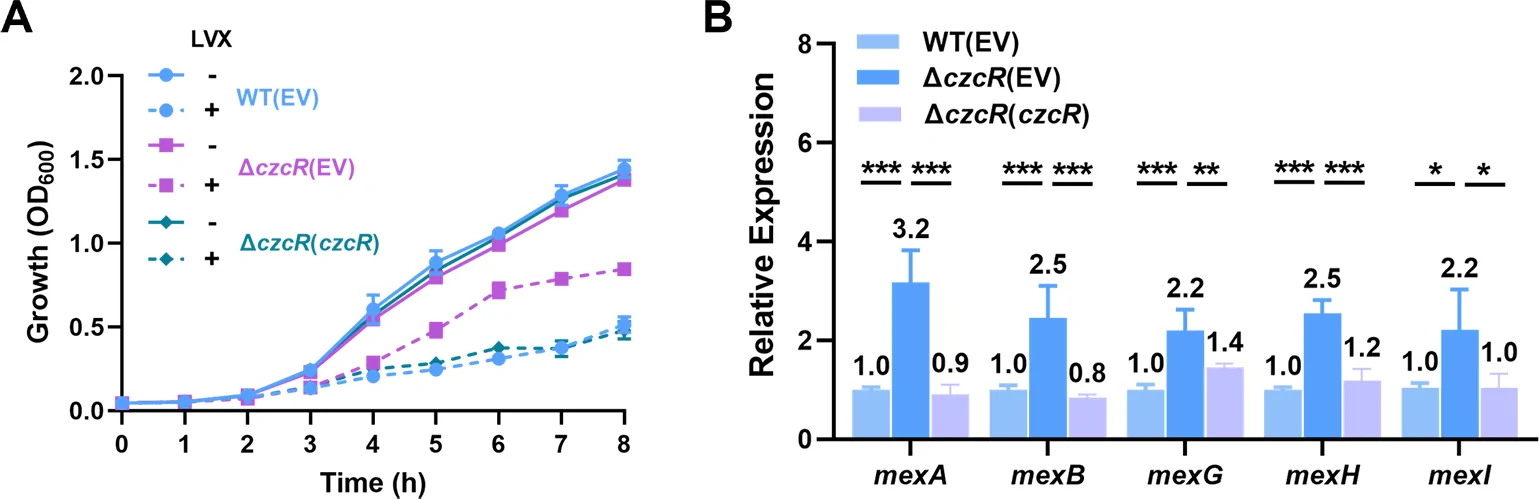
CzcR negatively regulates the resistance level to levofloxacin and the expression of *mexAB* and *mexGHI* in *P. aeruginosa*. (**A**) Growth of the PAO1 WT(EV), Δ*czcR*(EV), and Δ*czcR*(*czcR*) strains in the absence or presence of levofloxacin (LVX). (B) Relative expression of the *mexAB* and *mexGHI* genes in Δ*czcR*(EV) and Δ*czcR*(*czcR*) strains compared to those in the PAO1 WT(EV) strain. EV: empty vector which does not carry the *czcR* gene. Statistical significance is calculated based on the Student’s *t*-test (*P < 0.05; **P < 0.01; ***P < 0.001).

Our recent study showed that expression and activation of CzcR require the presence of an inducing signal Zn^2+^ (11); we therefore explored whether the TCS CzcS/CzcR influences the resistance of *P. aeruginosa* to other antibiotics in addition to carbapenems by culturing the *P. aeruginosa* strains in the presence of 0.5 mM ZnSO_4_. Levofloxacin, a commonly used antibiotic to treat *P. aeruginosa* infections from the class of fluoroquinolones (12), was first examined, and meropenem from the class of carbapenem was also examined as a control. It was shown that the loss of CzcR reduced the resistance level of *P. aeruginosa* to meropenem by two-fold MIC (Fig. S1A), which is consistent with the previous report that CzcR negatively regulates *oprD* to induce *P. aeruginosa* resistance to carbapenems (4). In contrast, we found that the MIC of levofloxacin was increased by two-fold from 0.5 to 1.0 μg/mL with the deletion of the *czcR* gene (Fig. S1A). Complemented expression of *czcR* in the Δ*czcR* mutant reduced the MIC value back to 0.5 µg/mL (Fig. S1A). This result showed that the presence of CzcR reduced the resistance level of *P. aeruginosa* to levofloxacin. To further confirm the role of CzcR in modulating levofloxacin resistance, we monitored the growth of PAO1 WT(EV), Δ*czcR*(EV), and Δ*czcR*(*czcR*) strains in the absence and presence of levofloxacin. Growth of these strains was similar in the absence of levofloxacin, while only the Δ*czcR*(EV) strain grow faster than other two strains in the presence of levofloxacin (Fig. 1A), confirming that CzcR negatively regulates levofloxacin resistance in *P. aeruginosa*.

*P. aeruginosa* encodes multiple efflux pumps such as MexAB-OprM, MexCD-OprJ, MexEF-OprN, MexGHI-OpmD, and MexXY, and they contribute significantly to the antibiotic resistance of *P. aeruginosa* (13, 14). To determine if any efflux pumps were repressed by CzcR, reverse transcription quantitative PCR (RT-qPCR) was performed to quantify their expression in the PAO1 WT and Δ*czcR* strains. As shown in Fig. S1B, upregulation of *mexAB* and *mexGHI* genes was observed in the Δ*czcR* mutant compared to the wild-type (WT) strain. Complementation with *czcR* in the Δ*czcR* strain significantly reduced the expression of *mexAB* and *mexGHI* genes (Fig. 1B). These results indicated that CzcR represses the expression of efflux pumps MexAB-OprM and MexGHI-OpmD. Deletion of *czcR* led to the upregulation of MexAB-OprM and MexGHI-OpmD, which possibly resulted in higher resistance level to levofloxacin.

### Development of a plasmid-based type I-F CRISPRi system (CSYi system) for targeted bacterial gene repression

**Fig 2:**
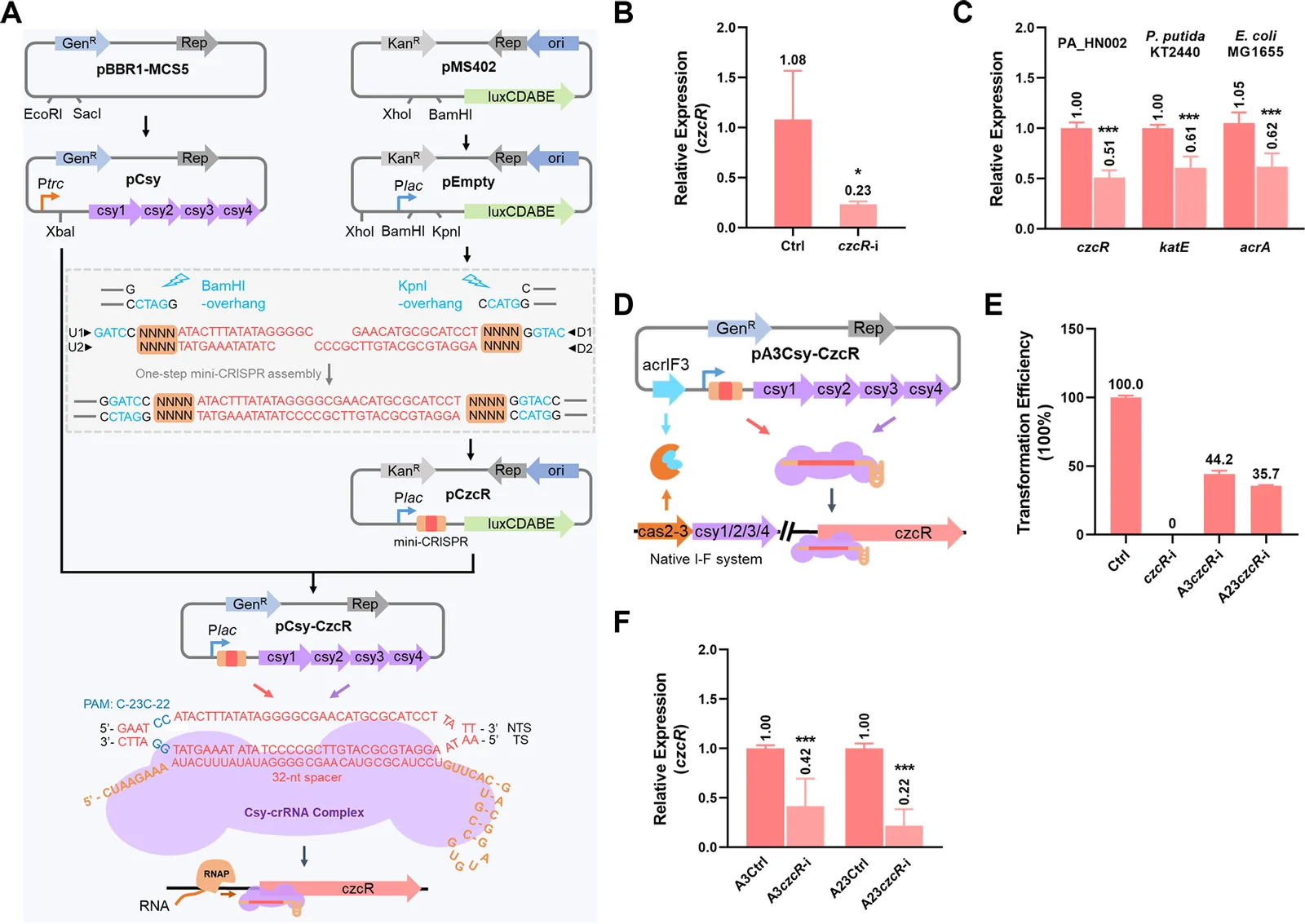
Development of a CSYi system for gene silencing. (**A**) Steps to construct plasmids for targeted gene repression (*czcR* as an example). The *csy* operon which contains *csy1*, *csy2*, *csy3*, and *csy4* genes was obtained from the genome of *P. aeruginosa* PA14. P*trc* promoter was obtained from the pACRISPR plasmid. The P*trc* promoter and *csy* operon were assembled between the EcoRI and SacI sites on the plasmid of pBBR1-MCS5, generating pCsy. P*lac* promoter together with its downstream BamHI and KpnI sites was obtained from pUC57 and inserted at the BamHI site of the plasmid pMS402, generating pEmpty. Oligonucleotides U1 and U2, D1 and D2 were *in vitro* phosphorylated and annealed to form double-stranded DNA fragments, respectively, and then assembled between the BamHI and KpnI sites of pEmpty, generating pCzcR which contained a mini-CRISPR to encode a crRNA targeting the promoter region of the *czcR* gene. NNNN indicated the 28-bp repeat sequence (5′-GTTCACTGCCGTATAGGCAGCTAAGAAA-3′) of the CRISPR array. 32-bp spacer sequence was highlighted in red. The P*lac* promoter and the mini-CRISPR were amplified together and inserted at the XbaI site of the pCsy plasmid, generating pCsy-CzcR. A diagram of the crRNA-guided Csy-crRNA complex binding to the promoter of the *czcR* gene was shown. (**B**) Relative expression of the *czcR* gene in PAO1 strains containing the pCsy-CzcR plasmid (*czcR*-i) or the control plasmid (Ctrl) which did not carry the mini-CRISPR. (**C**) Relative expression of the *czcR*, *katE*, *acrA* genes in PA_HN002, *P. putida* KT2440, *E. coli* MG1655 strains containing the CSYi plasmids targeting the *czcR*, *katA*, *acrA* genes, respectively, or the control plasmid (Ctrl), which did not carry mini-CRISPR. (**D**) Diagram of the pA3Csy-CzcR plasmid and its working mechanism to achieve targeted gene silencing in strains containing native type I-F CRISPR-Cas systems. (**E**) Relative recovery rate of the PA14 strain by electroporation of the control plasmid (Ctrl), the pCsy-CzcR plasmid (*czcR*-i), the pA3Csy-CzcR plasmid (A3*czcR*-i), and the pA23Csy-CzcR plasmid (A23*czcR*-i). (**F**) Relative expression of the *czcR* gene in PA14 strains containing the pA3Csy-CzcR plasmid (A3*czcR*-i) or the control plasmid (A3Ctrl), which did not carry the mini-CRISPR, and PA14 strains containing the pA23Csy-CzcR plasmid (A23*czcR*-i) or the control plasmid (A23Ctrl), which did not carry the mini-CRISPR. Statistical significance is calculated based on the Student’s *t*-test (*P < 0.05; ***P < 0.001).

We then moved to develop a new type I-F CRISPRi system (CSYi system) that can work for various bacterial hosts and sought to harness it to investigate whether the upregulation of MexAB-OprM and MexGHI-OpmD systems in the Δ*czcR* strain contributed to its higher levofloxacin resistance than the WT strain. The *czcR* gene was selected as a target gene for the establishment of the CSYi system. We initially employed two plasmids pCsy and pCzcR to, respectively, carry the *csy* genes (*csy1*, *csy2*, *csy3*, and *csy4*) and the CRISPR RNA (crRNA)-encoding element named mini-CRISPR (Fig. 2A). The *csy* operon was amplified from the genome of *P. aeruginosa* PA14, and the mini-CRISPR was designed to target the region close to the start codon, which was demonstrated as a region to cause the most effective gene repression (9). For *czcR*, a mini-CRISPR carrying a 32-bp spacer sequence that is identical to the 32-bp protospacer sequence preceded by the PAM “C-23C-22” in the *czcR* promoter region was designed and generated in one step with four oligonucleotides (U1, U2, D1, and D2) as shown in Fig. 2A. When we introduced pCsy and pCzcR into PAO1, colonies were recovered but they grew poorly in the liquid culture. This was possibly ascribed to the severe pressure from two antibiotics or the incompatibility of these two plasmids. To solve this problem, we simplified the system by assembling the *csy* operon and the mini-CRISPR into a single plasmid. A single CSYi plasmid pCsy-CzcR (*czcR*-i) was then constructed by incorporating the mini-CRISPR into the pCsy plasmid, which enabled the co-transcription of the *csy* operon and the mini-CRISPR driven by the same promoter P*lac* (Fig. 2A). After introducing *czcR*-i into PAO1, recovered colonies could grow normally (Fig. S2), meaning that the CSYi system is not cytotoxic. All the *csy* genes were expressed ordinarily with a similar level in the PAO1 strain (Fig. S3). Next, the expression of the target gene *czcR* in the PAO1 strain harboring *czcR*-i was measured, which showed a 77% reduction compared to the strain harboring the pCsy-Empty plasmid (Ctrl) which only carried the *csy* genes but without the mini-CRISPR (Fig. 2B). This result indicated that the single plasmid-based CSYi system is functional to repress target genes with simplicity.

We further examined the applicability of the CSYi system in a clinical *P. aeruginosa* isolate PA_HN002 (15) and strains from other bacterial species such as *Pseudomonas putida* KT2440 and *Escherichia coli* MG1655. *csy* genes were confirmed to express normally in all the strains (Fig. S3). Then, *czcR*, *katE*, and *acrA* genes were selected, respectively, for evaluating the activity of CSYi system in PA_HN002, *P. putida* KT2440, and *E. coli* MG1655. RT-qPCR results showed that expression levels of these genes in the respective strains were significantly reduced to 51%, 61%, and 62% compared to their control groups (Fig. 2C). These results displayed that the CSYi system is also functional in clinical *P. aeruginosa* strains and other bacterial species.

### Anti-CRISPR proteins enable the CSYi system to work in strains encoding native type I-F CRISPR-Cas systems

Anti-CRISPR proteins (Acr) are a group of CRISPR-Cas antagonists encoded by mobile genetic elements to inhibit CRISPR-Cas immunity (16). To date, 24 Acrs (AcrIF1–AcrIF24) that inactivate type I-F CRISPR-Cas systems were discovered (17). Among them, AcrIF3 was demonstrated to prevent the recruitment of Cas2-3 to the targeting site, and AcrIF23 was found to inhibit the nuclease activity of Cas2-3 (18, 19). Therefore, both Acrs could be promisingly employed to re-activate type I-F CRISPRi in the strains encoding native type I-F CRISPR-Cas systems. To achieve CSYi-based gene repression in strains containing native type I-F CRISPR-Cas systems, we next assembled *acrIF3* and *acrIF23* genes separately to the pCsy-CzcR plasmid, generating pA3Csy-CzcR (A3*czcR*-i) and pA23Csy-CzcR (A23*czcR*-i) (Fig. 2D). PA14 was selected to test the functionality of the improved CSYi system. Compared to no recovery after the introduction of *czcR*-i into PA14, colonies were obtained after the transformation of A3*czcR*-i or A23*czcR*-i (Fig. 2E), suggesting that both Acrs prevented the CRISPR-Cas-mediated genome cleavage. Then, we conducted RT-qPCR to quantify the expression of the *czcR* gene in the PA14 strains containing the plasmid A3*czcR*-i or A23*czcR*-i. It showed that the expression of *czcR* was reduced to 42% and 22%, respectively (Fig. 2F), implying the success of using both AcrIF3 and AcrIF23 to achieve CSYi-based gene silencing in strains containing active type I-F CRISPR-Cas systems.

### Increased levofloxacin resistance in Δ*czcR* is mainly ascribed to the upregulation of MexAB-OprM

**Fig 3:**
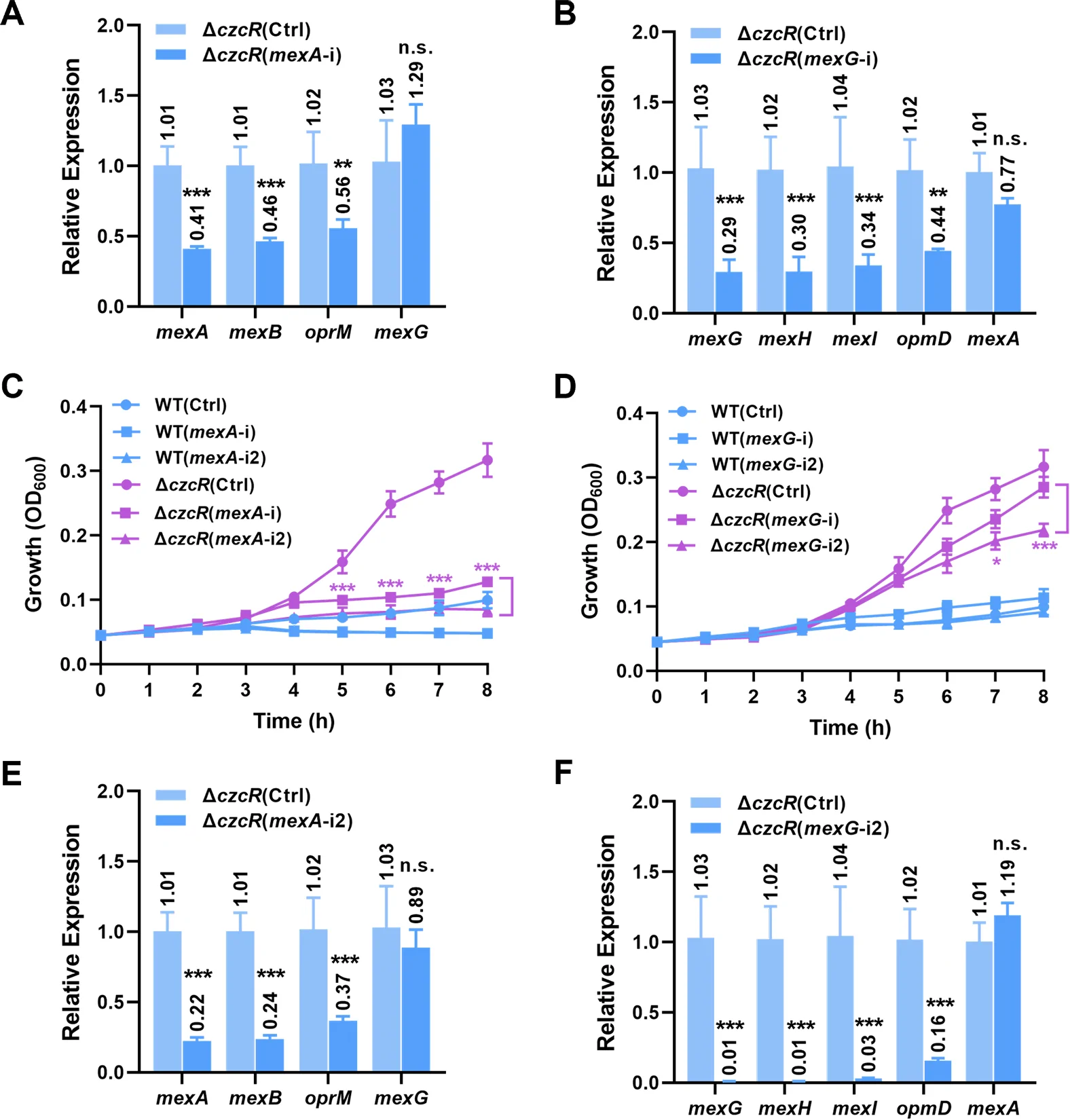
CSYi-targeted repression of MexAB-OprM abolishes the elevated levofloxacin resistance in Δ*czcR*. (**A**) Relative expression of the *mexAB-oprM* genes in the Δ*czcR* strains containing the pCsy-MexA plasmid (*mexA*-i) or the control plasmid (Ctrl), which did not carry mini-CRISPR. The *mexG* gene was selected as a negative control. (**B**) Relative expression of the *mexGHI-opmD* genes in the Δ*czcR* strains containing the pCsy-MexG plasmid (*mexG*-i) or the control plasmid (Ctrl), which did not carry mini-CRISPR. The *mexA* gene was selected as a negative control. (**C**) Growth of the PAO1 WT and Δ*czcR* strains containing the pCsy-MexA (*mexA*-i) plasmid, the pCsy-MexA2 (*mexA*-i2) plasmid, or the control plasmid (Ctrl), which did not carry mini-CRISPR in the presence of levofloxacin. (**D**) Growth of the PAO1 WT and Δ*czcR* strains containing the pCsy-MexG (*mexG*-i) plasmid, the pCsy-MexG2 (*mexG*-i2) plasmid, or the control plasmid (Ctrl), which did not carry mini-CRISPR in the presence of levofloxacin. (**E**) Relative expression of the *mexAB-oprM* genes in the Δ*czcR* strains containing the pCsy-MexA2 plasmid (*mexA*-i2) or the control plasmid (Ctrl), which did not carry mini-CRISPR. The *mexG* gene was selected as a negative control. (**F**) Relative expression of the *mexGHI-opmD* genes in the Δ*czcR* strains containing the pCsy-MexG2 plasmid (*mexG*-i2) or the control plasmid (Ctrl), which did not carry mini-CRISPR. The *mexA* gene was selected as a negative control. Statistical significance is calculated based on the Student’s *t-*test (n.s., not significant; *P < 0.05; **P < 0.01; ***P < 0.001).

With the developed CSYi system, we then moved to investigate whether the higher expression levels of MexAB-OprM and MexGHI-OpmD efflux pumps in the Δ*czcR* mutant contributed to its higher resistance level to levofloxacin than the PAO1 WT strain. We constructed two CSYi plasmids (*mexA*-i and *mexG*-i) to repress the operons of *mexAB-oprM* and *mexGHI-opmD*, respectively, by targeting the sequences spanning the start codon of the *mexA* and *mexG* genes on the template strand (TS) (Fig. S4). As shown in Fig. 3A and B, the introduction of *mexA*-i and *mexG*-i into Δ*czcR* led to 44%–59% and 56%–71% repression of genes in the operons of *mexAB-oprM* and *mexGHI-opmD*, respectively, compared to the strain Δ*czcR*(Ctrl). As controls, expression levels of the *mexG* and *mexA* genes were not significantly influenced by introducing the *mexA*-i and *mexG*-i plasmids, respectively (Fig. 3A and B), showing the specificity of the CSYi system.

To evaluate whether the CSYi-repressed expression of efflux pumps MexAB-OprM and MexGHI-OpmD could reduce the level of levofloxacin resistance of Δ*czcR* back to the WT level, we incubated the Δ*czcR*(*mexA*-i) and Δ*czcR*(*mexG*-i) strains in the presence of levofloxacin and monitored their growth. Compared to the normal growth of the control strain Δ*czcR*(Ctrl), we found that repression of the MexAB-OprM efflux pump in Δ*czcR* almost abolished its growth (Fig. 3C). However, the Δ*czcR* mutant with repressed expression of MexGHI-OpmD displayed a slight growth defect compared with Δ*czcR*(Ctrl) (Fig. 3D). These results suggested that upregulation of MexAB-OprM was the key contributor to the increased resistance level to levofloxacin in Δ*czcR*. To exclude the possibility that the inhibited growth of the Δ*czcR*(*mexA*-i) strain was owing to the *mexA*-targeting CSYi system, we compared the growth of Δ*czcR*(*mexA*-i) and Δ*czcR*(Ctrl) in the absence of levofloxacin, which did not show a detectable difference between two strains (Fig. S5). These results together indicated that CzcR mainly represses the expression of MexAB-OprM and consequently leads to the increased susceptibility of *P. aeruginosa* to levofloxacin.

Previous studies showed that crRNA-guided cascade binding to TS and non-template strand (NTS) led to different efficiencies of gene repression (20 – 22). To further compare the repression efficiency of the CSYi system when it targets different strands, we designed two additional CSYi plasmids *mexA*-i2 and *mexG*-i2 to target the NTS at locations close to the start codon as well (Fig. S4). Results exhibited that the CSYi system targeting the NTS led to a higher repression level of both efflux operons (Fig. 3E and F). *mexA*-i2 caused 63%–77% reduction of *mexAB-oprM* expression in Δ*czcR*, and *mexG*-i2 caused 84%–99% reduction of *mexGHI-opmD* expression, which almost fully inhibited the expression of *mexGHI-opmD*. Consistent with the lower expression level of the MexAB-OprM efflux pump, Δ*czcR*(*mexA*-i2) displayed a further inhibited growth compared to the Δ*czcR*(*mexA*-i) strain and showed an overlapped growth curve as the WT(Ctrl) strain in the presence of levofloxacin (Fig. 3C). In addition, Δ*czcR*(*mexG-*i2) also showed a further inhibited growth (Fig. 3D), suggesting that the efflux pump MexGHI-OpmD confers resistance of *P. aeruginosa* to levofloxacin as well but with a moderate effect when its expression is almost fully blocked. Together, these results showed that CSYi targeting the NTS led to more effective gene repression, and the resistance levels to levofloxacin were positively correlated to the expression levels of the *mex* genes.

### CzcR regulates *mex* genes expression by directly interacting with their promoters

**Fig 4:**
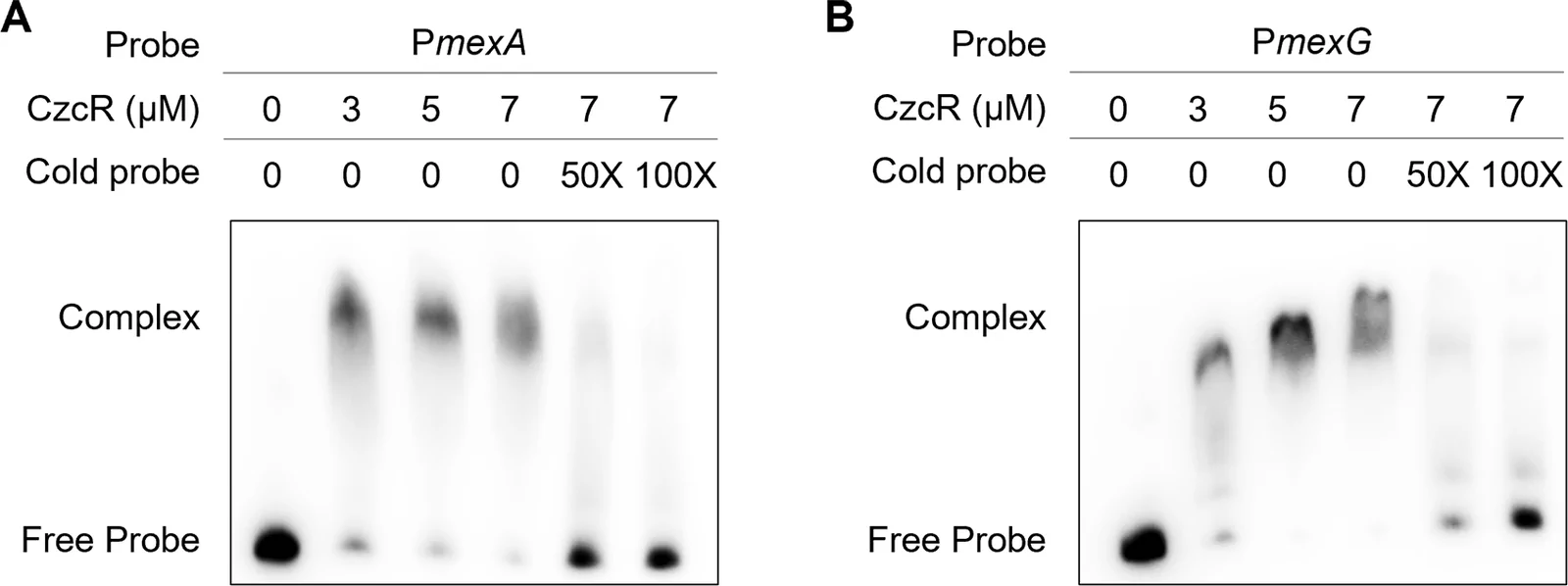
CzcR directly binds to the promoters of the *mexAB-oprM* and *mexGHI-opmD* operons. EMSA examination shows the interactions between CzcR and the promoters of *mexAB-oprM* (**A**) and *mexGHI-opmD* (**B**).

Expression of *mexAB-oprM* was reported to be governed directly or indirectly by a set of regulators such as MexR, MexT, and NalD (23). We sought to know whether CzcR repressed *mexAB-oprM* by changing the expression of these regulators. RT-qPCR showed that these regulator genes were expressed at a similar level between PAO1 WT and Δ*czcR* strains (Fig. S6), suggesting that they might not contribute to the upregulation of *mexAB-oprM* in Δ*czcR*. Given that CzcR itself is a transcription factor and direct binding of CzcR with various promoters was reported (24), we then investigated if CzcR could recognize the promoter of *mexAB-oprM* and thus regulate its expression directly. Electrophoretic mobility shift assay (EMSA) was performed after the CzcR protein and the 168-bp promoter sequence (probe) of *mexAB-oprM* were purified. As shown in Fig. 4A, CzcR and the promoter of *mexAB-oprM* were bound in a complex. The amount of shifted biotin-labeled probe was decreased in the presence of 50- and 100-fold greater concentrations of the unlabeled probe (cold probe) (Fig. 4A). We also used a promoter of a housekeeping gene *recA* to serve as a negative control and did not observe any interaction between the negative control and CzcR (Fig. S7). These results suggested that CzcR controls the expression of *mexAB-oprM* by directly interacting with its promoter. Similarly, the interaction between CzcR and the promoter of *mexGHI-opmD* was observed (Fig. 4B). These results showed that CzcR represses *mexAB-oprM* and *mexGHI-opmD* by directly interacting with their promoters.

### Repression of MexAB-OprM by CzcR results in decreased resistance to amikacin

**Fig 5:**
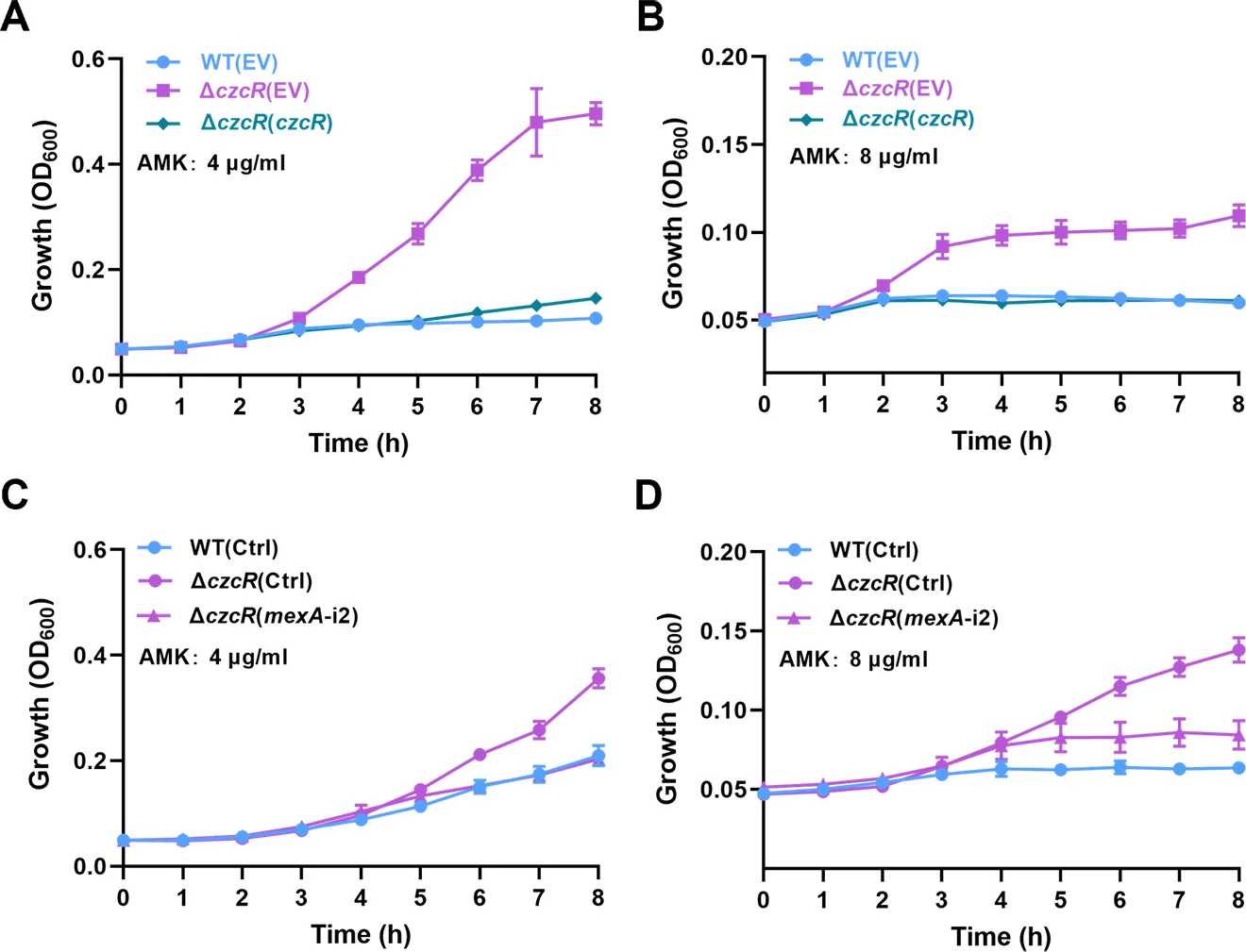
CzcR negatively regulates the resistance level of *P. aeruginosa* to amikacin. (**A and B**) Growth of the PAO1 WT(EV), Δ*czcR*(EV), and Δ*czcR*(*czcR*) strains in the presence of 4 µg/mL of amikacin (AMK) (**A**) or 8 µg/mL of AMK (**B**). (**C and D**) Growth of the Δ*czcR* strains containing the pCsy-MexA2 (*mexA*-i2) plasmid or the control plasmid (Ctrl), which did not carry mini-CRISPR in the presence of 4 µg/mL of AMK (**C**) or 8 µg/mL of AMK (**D**).

MexAB-OprM is a well-studied efflux pump extruding a broad spectrum of antibiotics in *P. aeruginosa*, and its overexpression was demonstrated to be associated with the induced bacterial resistance to multiple antimicrobial agents including most clinically prescribed antibiotics (25). We surmised that the upregulated expression of *mexAB-oprM* in the Δ*czcR* mutant might enhance the resistance of the Δ*czcR* mutant to other antibiotics in addition to levofloxacin. Aminoglycoside antibiotic amikacin was then evaluated, and as expected, we found that the Δ*czcR* mutant showed two-fold higher MIC of amikacin compared to the WT strain and complemented expression of *czcR* in the Δ*czcR* mutant abolished the increased MIC of amikacin (Fig. S1A). Consistent with the result of the MIC assay, Δ*czcR*(EV) was able to grow in the presence of 4 µg/mL and 8 µg/mL amikacin, while the growth of the WT(EV) and Δ*czcR*(*czcR*) strains was fully inhibited (Fig. 5A and B). Increased growth of the Δ*czcR* mutant in the presence of amikacin was abolished when the expression of *mexAB-oprM* was reduced by the CSYi plasmid *mexA*-i2 (Fig. 5C and D). All these data together confirmed that CzcR is a repressor of *mexAB-oprM* and inhibits multidrug resistance in *P. aeruginosa*.

## DISCUSSION

Genetic modification is generally difficult and sometimes impracticable in many bacterial strains owing that the intrinsic recombination capacity of most bacterial strains is poor. CRISPRi provides an alternative tool for functional genetic analysis by simply preventing the recruitment of transcription factors and RNA polymerase (RNAP) or blocking the movement of RNAP with crRNA-guided targeting of Cas proteins at a specific genomic site (26). Current applications of CRISPRi in bacteria are mainly based on the type II Cas9 system. However, expression of the Cas9 or the catalytically inactive dCas9 proteins in some bacterial species is cytotoxic and detrimental to cell growth (27, 28). Although type I systems are relatively complicated in structure, subtypes such as the I-B and I-F CRISPR-Cas systems were demonstrated to have higher specificity of genome targeting and efficiency of genome editing compared to the widely used type II Cas9 system in multiple cell types (9, 29). Based on the type I-F CRISPR-Cas system, in this study, we devised a single plasmid-based CSYi system that can be used to achieve desired repression of target genes in multiple bacterial hosts without detectable cellular toxicity. This system provides a new platform not only for studying antibiotic resistance but also for many other potential applications such as metabolic engineering and high-throughput screening in different bacterial hosts.

Although we showed the applicability of the CSYi system in diverse bacterial species, the extent of repression varied a lot among different genes and hosts. For example, it was shown that CSYi reduced the expression of *czcR* by 77% in *P. aeruginosa* PAO1, while it only caused a 49% reduction of *czcR* expression in another *P. aeruginosa* strain PA_HN002 despite *csy* genes in PA_HN002 were expressed at a much higher level than that in the PAO1 strain (Fig. S3). Owing that PA_HN002 is a clinical isolate that produces substantially higher production of the second messenger cyclic di-GMP and displays greater capability of biofilm formation than PAO1 (15), different genetic backgrounds might influence the activity of the CSYi system. In other bacterial strains such as *P. putida* KT2440 and *E. coli* MG1655, we also observed about 40% repression of target genes by the CSYi system. Lower efficiency of gene repression might be caused by multiple factors. In addition to the activity of the CSYi system, the optimal position of the target sequence is also important for efficient gene repression. However, the selection of target sequences depends on the availability of protospacer adjacent motifs (PAMs). Different locations of PAM sequences and their distance to the promoter or coding region of a target gene might impact the repression efficiency (20). Moreover, the importance of genes in bacterial growth is another factor influencing gene repression efficiency. It was previously observed that the endogenous type I-E CRISPRi system can reduce the expression level of *edd* by nearly 90% but less than 60% for *gnd* which is important for bacterial growth (30).

As a ubiquitous adaptive immune system, type I CRISPR-Cas systems are the most abundant CRISPR-Cas system and are widely distributed in many bacterial strains (31). However, current exploitations of the type I CRISPR-Cas system for gene silencing are very limited and mainly based on the native systems, which require the pre-deletion of the Cas nuclease genes from the genome. A universal type I CRISPRi system that can be applied to the strains with native CRISPR-Cas systems was not available. By incorporating the AcrIF3 and AcrIF23 proteins to specifically prevent genome cleavage by the Cas2-3 nuclease, the CSYi system we devised in this study is successfully improved to achieve gene silencing in the strains encoding native type I-F CRISPR-Cas systems. With the continuous discovery of Acrs that inactivate different subtypes of type I CRISPR-Cas systems, this study also shows the possibility of harnessing the abundant native type I CRISPR-Cas systems for targeted transcriptional modulation in genetically recalcitrant bacterial strains by incorporating Acrs.

Using EMSA assay and the newly developed gene-silencing system, we demonstrated that CzcR inhibits the expression of efflux pump genes *mexAB-oprM* and *mexGHI-opmD* by directly binding to the promoters, and the reduced expression of MexAB-OprM further resulted in the decreased resistance level of *P. aeruginosa* to multiple antibiotics. CzcR is a response regulator from the Zn^2+^-responsive TCS CzcS/CzcR, which has been reported to modulate carbapenem resistance and several other virulence-associated traits such as quorum sensing signaling, PYO biosynthesis, biofilm formation, and swimming motility in *P. aeruginosa* (11, 24, 32). Same as the *mexAB-oprM* and *mexGHI-opmD* efflux pump operons, CzcR regulates other genes such as the metal efflux pump gene operon *czcCBA*, porin-encoding gene *oprD*, quorum sensing gene *lasI*, PYO biosynthetic gene cluster *phz*, and flagellar biosynthetic genes by directly binding to their promoters (11, 24). A 16-bp motif GAAAC-N_6_-GTAAT was identified as the conserved CzcR-binding motif based on the top 10 enriched peak sequences from a chromatin immunoprecipitation sequencing (ChIP-Seq) result (33). However, we did not find this motif in the promoters of *mexAB-oprM* and *mexGHI-opmD*, which means that the binding motif of CzcR might not be restricted to GAAAC-N_6_-GTAAT and physiological roles of the TCS CzcS/CzcR remains largely underestimated.

Consistent with our recent findings showing that expression and activation of CzcR required the presence of the inducing signal Zn^2+^ (11), we found that deletion of *czcR* did not influence the expression of *mexAB* and other *mex* genes without the supplementation of ZnSO_4_ in the growth medium (Fig. S8). Therefore, supplementation of Zn^2+^ is required to activate CzcR for the repression of the efflux pump MexAB-OprM as well as the reduced resistance level of *P. aeruginosa* to different antibiotics. As a matter of fact, excessive Zn^2+^ release from intracellular storages to accumulate within phagosomes, lungs, or other tissues is one of the host defense strategies to intoxicate invading pathogens (34, 35). Toxicity of Zn^2+^ to bacteria is mainly ascribed to its capability of inducing protein mismetallation due to its top position in the Irving-Williams series, which frequently disorders other metal homeostatic networks or central metabolisms (36 – 38). For instance, it was reported that Zn^2+^ competitively binds to the pneumococcal surface adhesin A (PsaA) and then inhibits the uptake of manganese in *Streptococcus pneumoniae* (39). Findings in this study expanded our understanding of the activity of Zn^2+^, which serves as a signal to repress the expression of MexAB-OprM and consequently sensitize *P. aeruginosa* to levofloxacin and amikacin. Thus, our study suggested a promising strategy to combat *P. aeruginosa* infections with the combination of levofloxacin or amikacin and Zn^2+^-based antimicrobials.

## MATERIALS AND METHODS

### Bacterial strains, plasmids, primers, and growth conditions

Bacterial strains, plasmids and primers used in this study are summarized in Table S1. LB broth base and LB agar (Invitrogen) were used to prepare the medium for bacterial culture. Antibiotics were supplemented in the medium for plasmid propagation during bacterial growth: gentamicin, 50 µg/mL; kanamycin, 50 µg/mL; and ampicillin, 100 µg/mL. When necessary, different concentrations were added as specified.

### Plasmids construction for the two-plasmid and one-plasmid CSYi system

The *czcR* gene was selected as an example to show the construction procedures of plasmids used for CSYi gene repression. Primers used for plasmid construction were listed in Table S1. pCsy was generated by assembling the P*trc* promoter from pACRISPR (40) and the *csy* operon from the PA14 genome at EcoRI and SacI sites of the pBBR1-MCS5 plasmid using the ClonExpress II One Step Cloning Kit (Vazyme). pEmpty was generated by inserting a P*lac* promoter as well as the MCS region containing BamHI and KpnI sites from pUC57 into the XhoI and BamHI sites of the pMS402 plasmid. The original BamHI site in pMS402 was removed after the assembly. A 32-bp sequence preceded by the PAM “C-23C-22” located upstream of the *czcR* gene was selected as the protospacer for CSYi targeting. Four oligonucleotides (U1, U2, D1, and D2) containing the CRISPR repeat sequence and partial spacer sequence were synthesized by Sangon Biotech (China). After *in vitro* phosphorylation using the T4 Polynucleotide Kinase (New England Biolabs), U1 and U2, D1 and D2 were annealed to form two dsDNA fragments. Two fragments were then assembled into the pEmpty plasmid, which was pre-digested with BamHI and KpnI using the Quick Ligation Kit (New England Biolabs), generating pCzcR. The P*lac* promoter and assembled mini-CRISPR in pCzcR were amplified together and ligated into the pCsy plasmid, which was pre-treated with XbaI, yielding the pCsy-CzcR plasmid. Acr genes *acrIF3* and *acrIF23* were synthesized by Sangon Biotech (China). They were assembled, separately, into pEmpty at the XhoI site, generating two plasmids pEmpty-A3 and pEmpty-A23. When the target strain contained a native type I-F CRISPR-Cas system, these two plasmids were used for mini-CRISPR assembly. Then, the Acr gene, P*lac* promoter, and the mini-CRISPR were amplified together and inserted at the XbaI site of the pCsy plasmid.

### Preparation of electrocompetent cells and plasmid electroporation

A single bacterial colony was inoculated into 5 mL LB medium and grown at 37°C for approximately 12 hours with 220 rpm agitation. Two millliliters of bacterial culture was harvested by centrifugation at 16,000 × *g* for 2 minutes. Cell pellet was washed twice with 300 mM sucrose. After washing, 0.2 mL of 300 mM sucrose was used to resuspend the cell pellet and then electrocompetent cells were obtained. For electroporation of the pCsy-CzcR plasmid, 0.5 µg plasmid was mixed with 0.2 mL electrocompetent cells and subject to electroporation at 2.5 kV. One milliliter of LB medium was then added and the cells were recovered at 37°C for 1 hour with 220 rpm agitation. After recovering, bacterial cells were spread on LB plates containing 50 µg/mL gentamicin. Plates were incubated at 37°C for *P. aeruginosa* and 30°C for *P. putida*.

### MIC measurement

MIC was measured in 96-well plates following the procedures as described previously (41). Overnight culture was diluted and approximately 5.0 × 10^7^ CFU/mL cells were added in each well containing antibiotics with serially diluted concentrations ranging from 128 to 0.25 µg/mL. Plates were incubated at 37°C for 16 hours. The MIC value was determined as the lowest concentration of antibiotics that led to no visible cell growth in the well.

### Growth curve measurement

Single colonies of *P. aeruginosa* strains were inoculated in LB broth and grown overnight at 37°C with 220 rpm agitation. Overnight culture was 1:50 diluted into 5 mL fresh LB medium with the supplementation of 0.5 mM ZnSO_4_ and grown for 6 hours. After adjusting the cell number in each culture to approximately 1 × 10^7^ CFU/mL, levofloxacin or amikacin was added with indicated concentrations into the medium when necessary. Cell growth was monitored by measuring the OD_600_ value every 1 hour and then the growth curve was plotted. The result was displayed as the mean of biological triplicates.

### Reverse transcription-quantitative PCR

Overnight culture of bacterial strains was 1:50 diluted into 5 mL of fresh LB medium with the supplementation of 0.5 mM ZnSO_4_ and generally grown for 6 hours. 0.5 mL of bacterial culture was harvested, and total RNA was extracted using the Eastep Super Total RNA Extraction Kit (Promega) following the manufacturer’s instructions. cDNA was reverse transcribed using TransScript OneStep gDNA Removal and cDNA Synthesis SuperMix (TransGen). qPCR was performed using the ChamQ Universal SYBR qPCR Master Mix (Vazyme) in an ABI QuantStudioTM6 Flex system. The 2^-ΔΔCt^ method was used to calculate the relative expression of the target genes (42). The result was displayed as the mean of biological triplicates.

### CzcR purification and electrophoretic mobility shift assay

Protein purification was performed as previously described (38). *E. coli* BL21(DE3) containing the pET28a-*czcR* plasmid was incubated till OD_600_ of 0.6–0.8. Expression of the His_6_-tagged CzcR was induced by 0.5 mM isopropyl-β-d-thiogalactoside (IPTG) at 18°C for 16 hours. Purification of the His_6_-tagged CzcR protein was conducted using a Ni^2+^-affinity column. For EMSA, promoter fragments amplified from the PAO1 genome were biotin-labeled using the Biotin 3′-end DNA Labeling Kit (Thermo Fisher). Different amounts of the purified CzcR protein were incubated with the biotin-labeled DNA fragments (and cold probes) at 25°C for 30 minutes. The CzcR-DNA mixture was then subject to polyacrylamide gel electrophoresis (PAGE) in 0.5× Tris-Borate-EDTA (TBE) buffer at 75 V for 2 hours. The LightShift Chemiluminescent EMSA Kit (Thermo Fisher) was used to test the interaction between CzcR and promoter fragments according to the manufacturer’s instructions.

## SUPPLEMENTAL MATERIAL

Supplemental material spectrum.01123-23-s0001.pdf Fig. S1-S8 and Table S1.

ASM does not own the copyrights to Supplemental Material that may be linked to, or accessed through, an article. The authors have granted ASM a non-exclusive, world-wide license to publish the Supplemental Material files. Please contact the corresponding author directly for reuse.

## References

[1] Lee J , Zhang L . 2015. The hierarchy quorum sensing network in Pseudomonas aeruginosa. Protein Cell 6:26–41. doi:10.1007/s13238-014-0100-x 25249263 PMC4286720

[2] Moradali MF , Ghods S , Rehm BHA . 2017. Pseudomonas aeruginosa lifestyle: a paradigm for adaptation, survival, and persistence. Front Cell Infect Microbiol 7:39. doi:10.3389/fcimb.2017.00039 28261568 PMC5310132

[3] Francis VI , Stevenson EC , Porter SL . 2017. Two-component systems required for virulence in Pseudomonas aeruginosa. FEMS Microbiol Lett. 364. doi:10.1093/femsle/fnx104 PMC5812489 28510688

[4] Perron K , Caille O , Rossier C , Van Delden C , Dumas J-L , Köhler T . 2004. Czcr-Czcs, a two-component system involved in heavy metal and carbapenem resistance in Pseudomonas Aeruginosa. J Biol Chem 279:8761–8768. doi:10.1074/jbc.M312080200 14679195

[5] Wang D , Chen W , Huang S , He Y , Liu X , Hu Q , Wei T , Sang H , Gan J , Chen H , Dove SL . 2017. Structural basis of Zn(II) induced metal detoxification and antibiotic resistance by histidine kinase Czcs in Pseudomonas aeruginosa. PLoS Pathog 13:e1006533. doi:10.1371/journal.ppat.1006533 28732057 PMC5540610

[6] Xu Z , Li M , Li Y , Cao H , Miao L , Xu Z , Higuchi Y , Yamasaki S , Nishino K , Woo PCY , Xiang H , Yan A . 2019. Native CRISPR-cas-mediated genome editing enables dissecting and sensitizing clinical multidrug-resistant P. aeruginosa. Cell Reports 29:1707–1717. doi:10.1016/j.celrep.2019.10.006 31693906

[7] Larson MH , Gilbert LA , Wang X , Lim WA , Weissman JS , Qi LS . 2013. CRISPR interference (CRISPRi) for sequence-specific control of gene expression. Nat Protoc 8:2180–2196. doi:10.1038/nprot.2013.132 24136345 PMC3922765

[8] Bikard D , Jiang W , Samai P , Hochschild A , Zhang F , Marraffini LA . 2013. Programmable repression and activation of bacterial gene expression using an engineered CRISPR-Cas system. Nucleic Acids Res. 41:7429–7437. doi:10.1093/nar/gkt520 23761437 PMC3753641

[9] Xu Z , Li Y , Cao H , Si M , Zhang G , Woo PCY , Yan A . 2021. A transferrable and integrative type I-F cascade for heterologous genome editing and transcription modulation. Nucleic Acids Res. 49:e94. doi:10.1093/nar/gkab521 34157103 PMC8450077

[10] van Belkum A , Soriaga LB , LaFave MC , Akella S , Veyrieras J-B , Barbu EM , Shortridge D , Blanc B , Hannum G , Zambardi G , Miller K , Enright MC , Mugnier N , Brami D , Schicklin S , Felderman M , Schwartz AS , Richardson TH , Peterson TC , Hubby B , Cady KC , Parkhill J . 2015. Phylogenetic distribution of CRISPR-Cas systems in antibiotic-resistant Pseudomonas Aeruginosa. mBio 6. doi:10.1128/mBio.01796-15 PMC4669384 26604259

[11] Liu Z , Xu Z , Chen S , Huang J , Li T , Duan C , Zhang LH , Xu Z . 2022. CzcR is essential for swimming motility in Pseudomonas aeruginosa during zinc stress. Microbiol Spectr 10:e0284622. doi:10.1128/spectrum.02846-22 36416561 PMC9769499

[12] Bassetti M , Vena A , Croxatto A , Righi E , Guery B . 2018. How to manage Pseudomonas Aeruginosa infections. DIC 7:1–18. doi:10.7573/dic.212527 PMC5978525 29872449

[13] Dreier J , Ruggerone P . 2015. Interaction of antibacterial compounds with RND efflux pumps in Pseudomonas aeruginosa *.* Front Microbiol 6:660. doi:10.3389/fmicb.2015.00660 26217310 PMC4495556

[14] Aendekerk S , Ghysels B , Cornelis P , Baysse C . 2002. Characterization of a new efflux pump, MexGHI-OpmD, from Pseudomonas aeruginosa that confers resistance to vanadium. Microbiology (Reading) 148:2371–2381. doi:10.1099/00221287-148-8-2371 12177331

[15] Lin S , Chen S , Li L , Cao H , Li T , Hu M , Liao L , Zhang LH , Xu Z . 2022. Genome characterization of a uropathogenic Pseudomonas Aeruginosa isolate PA_HN002 with cyclic di-GMP-dependent hyper-biofilm production. Front Cell Infect Microbiol 12:956445. doi:10.3389/fcimb.2022.956445 36004331 PMC9394441

[16] Davidson AR , Lu W-T , Stanley SY , Wang J , Mejdani M , Trost CN , Hicks BT , Lee J , Sontheimer EJ . 2020. Anti-CRISPRs: Protein inhibitors of CRISPR-Cas systems. Annu Rev Biochem 89:309–332. doi:10.1146/annurev-biochem-011420-111224 32186918 PMC9718424

[17] Pinilla-Redondo R , Shehreen S , Marino ND , Fagerlund RD , Brown CM , Sørensen SJ , Fineran PC , Bondy-Denomy J . 2020. Discovery of multiple anti-CRISPRs highlights anti-defense gene clustering in mobile genetic elements. Nat Commun 11:5652. doi:10.1038/s41467-020-19415-3 33159058 PMC7648647

[18] Ren J , Wang H , Yang L , Li F , Wu Y , Luo Z , Chen Z , Zhang Y , Feng Y . 2022. Structural and mechanistic insights into the inhibition of type I-F CRISPR-Cas system by anti-CRISPR protein AcrIF23. J Biol Chem 298:102124. doi:10.1016/j.jbc.2022.102124 35697070 PMC9270243

[19] Rollins MF , Chowdhury S , Carter J , Golden SM , Miettinen HM , Santiago-Frangos A , Faith D , Lawrence CM , Lander GC , Wiedenheft B . 2019. Structure reveals a mechanism of CRISPR-RNA-guided Nuclease recruitment and anti-CRISPR viral mimicry. Mol Cell 74:132–142. doi:10.1016/j.molcel.2019.02.001 30872121 PMC6521718

[20] Luo ML , Mullis AS , Leenay RT , Beisel CL . 2015. Repurposing endogenous type I CRISPR-Cas systems for programmable gene repression. Nucleic Acids Res. 43:674–681. doi:10.1093/nar/gku971 25326321 PMC4288209

[21] Rath D , Amlinger L , Hoekzema M , Devulapally PR , Lundgren M . 2015. Efficient programmable gene silencing by cascade. Nucleic Acids Res. 43:237–246. doi:10.1093/nar/gku1257 25435544 PMC4288158

[22] Chen Y , Cheng M , Song H , Cao Y . 2022. Type I-F CRISPR-PAIR platform for multi-mode regulation to boost extracellular electron transfer in Shewanella oneidensis. iScience 25:104491. doi:10.1016/j.isci.2022.104491 35712075 PMC9194131

[23] Suresh M , Nithya N , Jayasree PR , Vimal KP , Manish Kumar PR . 2018. Mutational analyses of regulatory genes, mexR, nalC, nalD and mexZ of mexAB-oprM and mexXY operons, in efflux pump hyperexpressing multidrug-resistant clinical isolates of Pseudomonas Aeruginosa *.* World J Microbiol Biotechnol 34:83. doi:10.1007/s11274-018-2465-0 29846800

[24] Dieppois G , Ducret V , Caille O , Perron K . 2012. The transcriptional regulator CzcR modulates antibiotic resistance and Quorum sensing in Pseudomonas aeruginosa. PLoS One 7:e38148. doi:10.1371/journal.pone.0038148 22666466 PMC3362554

[25] Poole K . 2011. Pseudomonas aeruginosa: resistance to the max. Front Microbiol 2:65. doi:10.3389/fmicb.2011.00065 21747788 PMC3128976

[26] Qi LS , Larson MH , Gilbert LA , Doudna JA , Weissman JS , Arkin AP , Lim WA . 2013. Repurposing CRISPR as an RNA-guided platform for sequence-specific control of gene expression. Cell 152:1173–1183. doi:10.1016/j.cell.2013.02.022 23452860 PMC3664290

[27] Cui Y , Dong H , Tong B , Wang H , Chen X , Liu G , Zhang D . 2022. A versatile Cas12K-based genetic engineering toolkit (C12KGET) for metabolic engineering in genetic manipulation-deprived strains. Nucleic Acids Res. 50:8961–8973. doi:10.1093/nar/gkac655 35920322 PMC9410911

[28] Walker JE , Lanahan AA , Zheng T , Toruno C , Lynd LR , Cameron JC , Olson DG , Eckert CA . 2020. Development of both type I-B and type II CRISPR/Cas genome editing systems in the cellulolytic bacterium Clostridium Thermocellum. Metab Eng Commun 10:e00116. doi:10.1016/j.mec.2019.e00116 31890588 PMC6926293

[29] Pyne ME , Bruder MR , Moo-Young M , Chung DA , Chou CP . 2016. Harnessing heterologous and endogenous CRISPR-Cas machineries for efficient Markerless genome editing in Clostridium. Sci Rep 6:25666. doi:10.1038/srep25666 27157668 PMC4860712

[30] Qin Z , Yang Y , Yu S , Liu L , Chen Y , Chen J , Zhou J . 2021. Repurposing the endogenous type I-E CRISPR/Cas system for gene repression in Gluconobacter oxydans WSH-003. ACS Synth Biol 10:84–93. doi:10.1021/acssynbio.0c00456 33399467

[31] Cameron P , Coons MM , Klompe SE , Lied AM , Smith SC , Vidal B , Donohoue PD , Rotstein T , Kohrs BW , Nyer DB , Kennedy R , Banh LM , Williams C , Toh MS , Irby MJ , Edwards LS , Lin C-H , Owen ALG , Künne T , van der Oost J , Brouns SJJ , Slorach EM , Fuller CK , Gradia S , Kanner SB , May AP , Sternberg SH . 2019. Harnessing type I CRISPR-Cas systems for genome engineering in human cells. Nat Biotechnol 37:1471–1477. doi:10.1038/s41587-019-0310-0 31740839

[32] Lee J-H , Kim Y-G , Cho MH , Lee J . 2014. Zno nanoparticles inhibit Pseudomonas aeruginosa biofilm formation and virulence factor production. Microbiol Res 169:888–896. doi:10.1016/j.micres.2014.05.005 24958247

[33] Fan K , Cao Q , Lan L . 2021. Genome-wide mapping reveals complex regulatory activities of BfmR in Pseudomonas aeruginosa Microorganisms 9:485. doi:10.3390/microorganisms9030485 33668961 PMC8025907

[34] Botella H , Peyron P , Levillain F , Poincloux R , Poquet Y , Brandli I , Wang C , Tailleux L , Tilleul S , Charrière GM , Waddell SJ , Foti M , Lugo-Villarino G , Gao Q , Maridonneau-Parini I , Butcher PD , Castagnoli PR , Gicquel B , de Chastellier C , Neyrolles O . 2011. Mycobacterial P1-type ATPases mediate resistance to zinc poisoning in human macrophages. Cell Host & Microbe 10:248–259. doi:10.1016/j.chom.2011.08.006 21925112 PMC3221041

[35] Ong CY , Gillen CM , Barnett TC , Walker MJ , McEwan AG . 2014. An antimicrobial role for zinc in innate immune defense against group A Streptococcus. J Infect Dis. 209:1500–1508. doi:10.1093/infdis/jiu053 24449444

[36] Braymer JJ , Giedroc DP . 2014. Recent developments in copper and zinc homeostasis in bacterial pathogens. Curr Opin Chem Biol 19:59–66. doi:10.1016/j.cbpa.2013.12.021 24463765 PMC4008645

[37] Ong CY , Walker MJ , McEwan AG . 2015. Zinc disrupts central carbon metabolism and capsule biosynthesis in Streptococcus pyogenes *.* Sci Rep 5:10799. doi:10.1038/srep10799 26028191 PMC4450579

[38] Xu Z , Wang P , Wang H , Yu ZH , Au-Yeung HY , Hirayama T , Sun H , Yan A . 2019. Zinc excess increases cellular demand for iron and decreases tolerance to copper in Escherichia coli J Biol Chem 294:16978–16991. doi:10.1074/jbc.RA119.010023 31586033 PMC6851343

[39] McDevitt CA , Ogunniyi AD , Valkov E , Lawrence MC , Kobe B , McEwan AG , Paton JC . 2011. A molecular mechanism for bacterial susceptibility to zinc. PLoS Pathog 7:e1002357. doi:10.1371/journal.ppat.1002357 22072971 PMC3207923

[40] Chen W , Zhang Y , Zhang Y , Pi Y , Gu T , Song L , Wang Y , Ji Q . 2018. CRISPR/Cas9-based genome editing in Pseudomonas aeruginosa and cytidine deaminase-mediated base editing in Pseudomonas species. iScience 6:222–231. doi:10.1016/j.isci.2018.07.024 30240613 PMC6137401

[41] Cao H , Xia T , Li Y , Xu Z , Bougouffa S , Lo YK , Bajic VB , Luo H , Woo PCY , Yan A . 2019. Uncoupled quorum sensing modulates the interplay of virulence and resistance in a multidrug-resistant clinical Pseudomonas Aeruginosa isolate belonging to the MLST550 clonal complex . Antimicrob Agents Chemother 63:e01944–18. doi:10.1128/AAC.01944-18 30670423 PMC6437519

[42] Schmittgen TD , Livak KJ . 2008. Analyzing real-time PCR data by the comparative CT method. Nat Protoc 3:1101–1108. doi:10.1038/nprot.2008.73 18546601

