# Supplementary material for: A type I-F CRISPRi system unveils the novel role of CzcR in modulating multidrug resistance of Pseudomonas aeruginosa
Source: Microbiol Spectr. 2023 Aug 30;11(5):e01123-23. doi: 10.1128/spectrum.01123-23 (PMC10581170; doi:10.1128/spectrum.01123-23)
Supplement: Supplemental material — Fig. S1-S8 and Table S1. [file spectrum.01123-23-s0001.pdf]

Supplemental Material

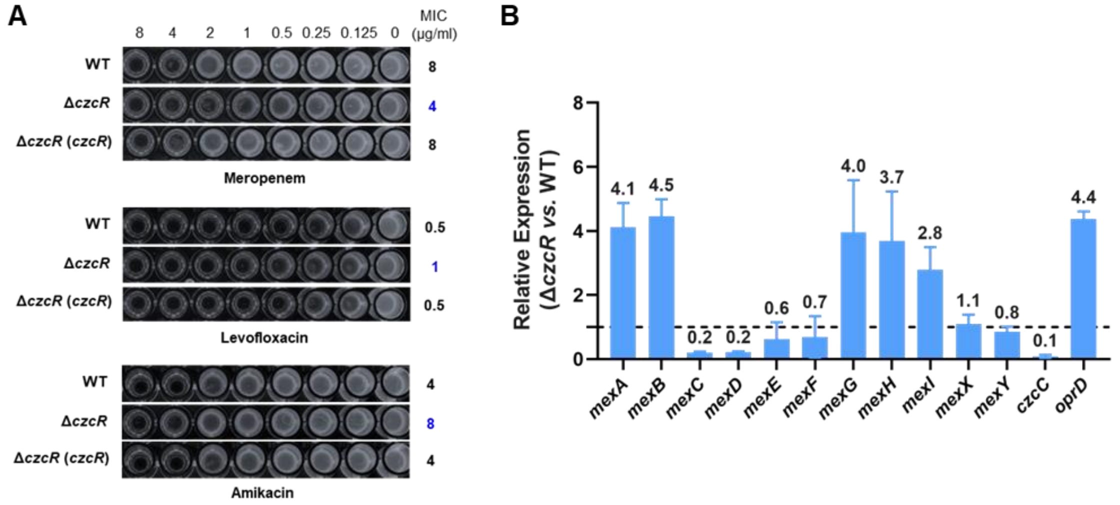

Figure S1. Examination of the role of CzcR in regulating antibiotic resistance and the expression of *mex* efflux genes in *P. aeruginosa*. (**A**) MICs of meropenem, levofloxacin and amikacin against the PAO1 WT, Δ*czcR* and Δ*czcR*(*czcR*) strains. Representative pictures of MIC measurements were shown. (**B**) Relative expression of the *mex* genes in the Δ*czcR* mutant compared to the PAO1 WT strain. The black dashed lines represent the same expression level of genes between Δ*czcR* and WT strains. When the fold change is larger than 1, it indicates higher expression of the gene in the Δ*czcR* mutant. When the fold change is smaller than 1, it indicates higher expression of the gene in the WT strain. *czcC* and *oprD* which are positively and negatively regulated by CzcR, respectively, were selected as controls.

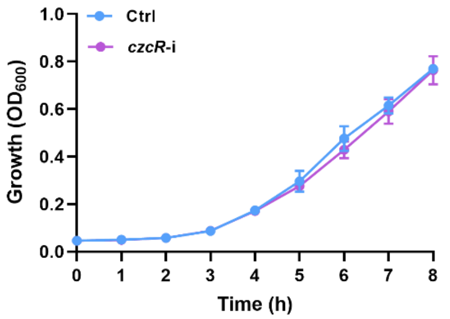

Figure S2. Growth of the PAO1 strains containing the pCsy-CzcR (*czcR*-i) plasmid or the pCsy-Empty plasmid (Ctrl).

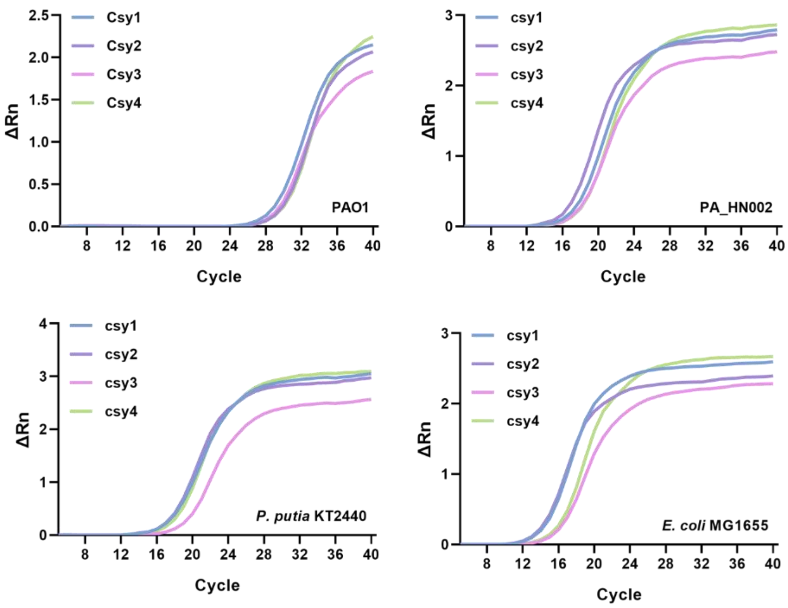

Figure S3. qPCR analysis for the expression of *csy* genes in the PAO1, PA_HN002, *P. putida* KT2440, and *E. coli* MG1655 strains which carried a pCsy plasmid with a specific mini-CRISPR.

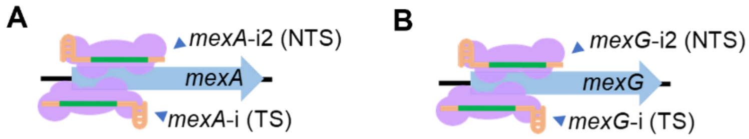

Figure S4. Diagrams showing the targeting patterns of the Csy-crRNA complex when the plasmids of *mexA*-i/*mexA*-i2 (**A**) or *mexG*-i/*mexG*-i2 (**B**) were introduced into PAO1 strains. TS: template strand, NTS: Non-template strand.

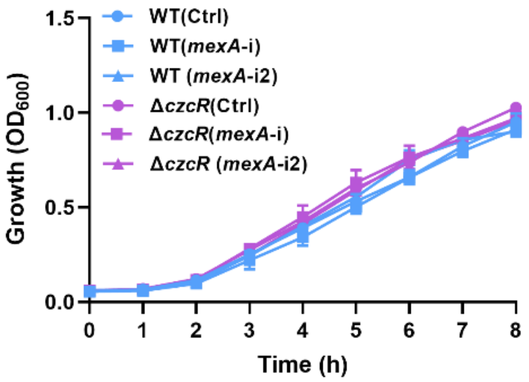

Figure S5. Growth of the PAO1 WT and Δ*czcR* strains containing the control plasmid (Ctrl), the pCsy-MexA (*mexA*-i) plasmid and the pCsy-MexA2 (*mexA*-i2) plasmid in the presence of 0.5 mM $ZnSO_4$ (Zn) but absence of levofloxacin.

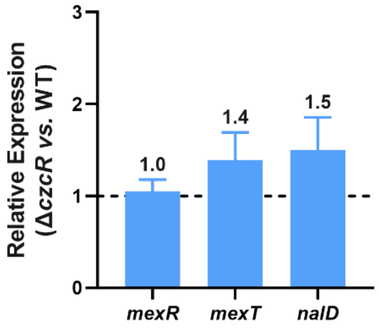

Figure S6. Relative expression of the *mexR*, *mexT* and *nalD* genes in the Δ*czcR* mutant compared to the PAO1 WT strain.

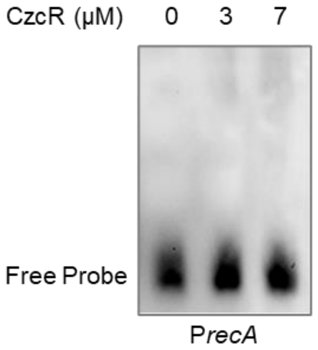

Figure S7. EMSA examination showing the incapable binding of CzcR to a 126-bp DNA fragment of the *recA* promoter which served as a negative control.

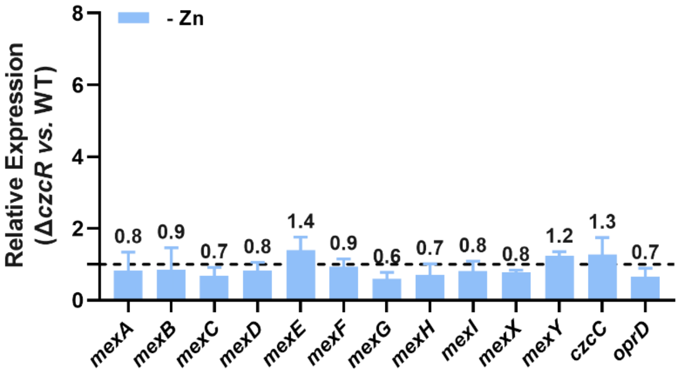

Figure S8. Relative expression of the *mex* genes in the Δ*czcR* mutant compared to the PAO1 WT strain when they were cultured in the absence of Zn.

Table S1. Bacterial strains, plasmids, and primers used in this study

1. Strains

Name	Description
DH5α	*E. coli* strain used for plasmid construction and propagation
BL21(DE3)	*E. coli* strain used for protein expression and purification
PAO1	*P. aeruginosa* PAO1 wild-type from lab collection
PAO1 Δ*czcR*	*P. aeruginosa* PAO1 with the deletion of *czcR* from lab collection
PAO1 Δ*czcR*(*czcR*)	PAO1 Δ*czcR* with the complementation of *czcR* from lab collection
PA14	*P. aeruginosa* PA14 from lab collection
PA_HN002	*P. aeruginosa* PA_HN002 from lab collection
P. putida KT2440	*P. putida* KT2440 from lab collection
E. coli MG1655	*E. coli* MG1655 from lab collection

2. Plasmids

Name	Description
pCsy	pBBR1-MCS5 carries the P*trc* promoter and Csy operon
pEmpty	pMS402 carries a P*lac* promoter
pEmpty-A3	pEmpty carries the *acrIF3* gene
pEmpty-A23	pEmpty carries the *acrIF23* gene
pCsy-CzcR(*czcR*-i)	CSYi system targets the *czcR* gene in PAO1
pCsy-CzcR(*katE*-i)	CSYi system targets the *katE* gene in *P. putida* KT2440
pCsy-CzcR(*acrA*-i)	CSYi system targets the *acrA* gene in *E. coli* MG1655
pA3Csy-CzcR	CSYi system targets the *czcR* gene in PA14
pA23Csy-CzcR	CSYi system targets the *czcR* gene in PA14
pCsy-MexA(*mexA*-i)	CSYi system targets the *mexA* gene (TS) in PAO1
pCsy-MexA(*mexA*-i2)	CSYi system targets the *mexA* gene (NTS) in PAO1
pCsy-MexG(*mexG*-i)	CSYi system targets the *mexG* gene (TS) in PAO1
pCsy-MexG(*mexG*-i2)	CSYi system targets the *mexG* gene (NTS) in PAO1
pET28a-*czcR*	For CzcR expression and purification
pUC-acrIF3	pUC57 carries a synthetic *acrIF3* gene
pUC-acrIF23	pUC57 carries a synthetic *acrIF23* gene

3. Primer sequences

Name	Sequence (5' to 3')	Description
Ptrc-F	GATAAGCTTGATATCGAATTCCATATGGTATACACTTTGCCCTTTACA	Amplify the P*trc* promoter from pACRISPR
Ptrc-R	CGAAAGCTGTGCTCCTGTTTAAACTCTAGAACTAGTCTTGCTATTTCTAGCTCTAAAAC	
Csy-F	ACTAGTTCTAGAGTTTAAACAGGAGCACAGCTTTCGAATGC	Amplify the *csy* operon from PA14
Csy-R	CTATAGGGCGAATTGGAGCTCCGGCCAGCAGCCCTGAAG	
Plac-F	GTCTTCACCTCGAGGCAGCTGGCACGACAGGTTTC	Amplify the P*lac* promoter from pUC57
Plac-R	GCGGCCGCAACTAGATTGTAAAACGACGGCCAGTG	
crRNA-F	AATAGCAAGACTAGTTCTAGACGAGGCCCTTTCGTCTTCAC	Amplify the P*lac* promoter and mini-CRISPR from pEmpty to pCsy
crRNA-R	TGCTCCTGTTTAAACTTGCGGCCGCAACTAGATTG	
pEmpty-veri-F	CCAGCTGGCAATTCCGA	Verify the construction of mini-CRISPR
pEmpty-veri-R	AATCATCACTTTCGGGAAAG	
Pcsy-veri-F	CGGCTCGTATAATGTGTG	Verify the insertion of mini-CRISPR into pCsy
Pcsy-veri-R	ACGAGTGCTCGCATTCGA	
AcrIF-F	AATAGCAAGACTAGTTCTAGACAGTGAGCGCAACGCAATT	Amplify acrIF3 and acrIF23 from pUC-acrIF3 and pUC-acrIF23
AcrIF-R	ACGAAAGGGCCTCGAGAAAATACCGCATCAGGCG	
CzcR-i-U1	GATCCGTTCACTGCCGTATAGGCAGCTAAGAAAATACTTTATATAGGGGC	mini-CRISPR construction for *czcR* gene
CzcR-i-U2	CTATATAAAGTATTTTCTTAGCTGCCTATACGGCAGTGAACG	

CzcR-i-D1	GAACATGCGCATCCTGTTCACTGCCGTATAGGCAGCTAAGAAAGGTAC	
CzcR-i-D2	CTTTCTTAGCTGCCTATACGGCAGTGAACAGGATGCGCATGTTCGCCC	
MexA-i-U1	GATCCGTTCACTGCCGTATAGGCAGCTAAGAAAGCTTTCGCTCATGAGGA	mini-CRISPR construction for *mexA* gene
MexA -i-U2	CATGAGCGAAAGCTTTCTTAGCTGCCTATACGGCAGTGAACG	
MexA -i-D1	CAACGCTATGCAACGGTTCACTGCCGTATAGGCAGCTAAGAAAGGTAC	
MexA -i-D2	CTTTCTTAGCTGCCTATACGGCAGTGAACCGTTGCATAGCGTTGTCCT	
MexA-i2-U1	GATCCGTTCACTGCCGTATAGGCAGCTAAGAAAAGCAGGGCCGGAACCAG	mini-CRISPR construction for *mexA* gene
MexA -i2-U2	TTCCGGCCCTGCTTTTCTTAGCTGCCTATACGGCAGTGAACG	
MexA -i2-D1	TACACGCATGGCTGGGTTCACTGCCGTATAGGCAGCTAAGAAAGGTAC	
MexA -i2-D2	CTTTCTTAGCTGCCTATACGGCAGTGAACCCAGCCATGCGTGTACTGG	
MexG-i-U1	GATCCGTTCACTGCCGTATAGGCAGCTAAGAAAGGATGGCGACCAGCACA	mini-CRISPR construction for *mexG* gene
MexG -i-U2	CTGGTCGCCATCCTTTCTTAGCTGCCTATACGGCAGTGAACG	
MexG -i-D1	AGGAACGACCCATGCGTTCACTGCCGTATAGGCAGCTAAGAAAGGTAC	
MexG -i-D2	CTTTCTTAGCTGCCTATACGGCAGTGAACGCATGGGTCGTTCCTTGTG	
MexG-i2-U1	GATCCGTTCACTGCCGTATAGGCAGCTAAGAAAAGTTGCTTTCGAGCGAG	mini-CRISPR construction for *mexG* gene
MexG -i2-U2	CTCGAAAGCAACTTTTCTTAGCTGCCTATACGGCAGTGAACG	
MexG -i2-D1	TTATCGATGAAGCGCGTTCACTGCCGTATAGGCAGCTAAGAAAGGTAC	
MexG -i2-D2	CTTTCTTAGCTGCCTATACGGCAGTGAACGCGCTTCATCGATAACTCG	
KatE-i-U1	GATCCGTTCACTGCCGTATAGGCAGCTAAGAAATTAAGACTGATGAGGAG	mini-CRISPR construction for *katE* gene
KatE-i-U2	TCATCAGTCTTAATTTCTTAGCTGCCTATACGGCAGTGAACG	
KatE-i-D1	AGCCGATCATGCCCAGTTCACTGCCGTATAGGCAGCTAAGAAAGGTAC	
KatE-i-D2	CTTTCTTAGCTGCCTATACGGCAGTGAACTGGGCATGATCGGCTCTCC	
AcrA-i-U1	GATCCGTTCACTGCCGTATAGGCAGCTAAGAAAAATTTGAAATCGGACAC	mini-CRISPR construction for *acrA* gene
AcrA-i-U2	CCGATTTCAAATTTTTCTTAGCTGCCTATACGGCAGTGAACG	
AcrA-i-D1	TCGAGGTTTACATATGTTCACTGCCGTATAGGCAGCTAAGAAAGGTAC	
AcrA-i-D2	CTTTCTTAGCTGCCTATACGGCAGTGAACATATGTAAACCTCGAGTGT	
recA-RT-F	CAACTGCCTGGTCATCTTCATC	qPCR
recA-RT-R	CGTAGAACTTCAGTGCGTTACC	
mexA-RT-F	GACGGTGACCCTGAATAC	qPCR
mexA-RT-R	GATCTGGTAGAGCTGCTG	
mexB-RT-F	CGAATACCTGCTGGAGAA	qPCR
mexB-RT-R	CCTTGAAGCTGAAGAAGTG	
mexC-RT-F	GACCTGCTGTTCCAGATC	qPCR
mexC-RT-R	GGTATCGAAGTCCTGCTG	
mexD-RT-F	TGATCTACCAGCAGTTCTC	qPCR
mexD-RT-R	GAGTTGAGCAGCGAATAG	
mexE-RT-F	GTCATCCCACTTCTCCTG	qPCR
mexE-RT-R	AATTCGTCCCACTCGTTC	
mexF-RT-F	GACCTACTTCGGCTTCTC	qPCR
mexF-RT-R	CTCGGACATCTGCTTGAT	
mexG-RT-F	AGCTGTTCGACTATCAGG	qPCR
mexG-RT-R	GACGATGAGGATGGTCAG	
mexH-RT-F	GTCACCTATACCGCCTAC	qPCR
mexH-RT-R	CTCAGGTTGATCTGTCCG	
mexI-RT-F	CATCTTCGCCTTCCAGTT	qPCR
mexI-RT-R	TTCTGCTTGATCTCTTCCAT	
mexX-RT-F	TCCGATCTACGTGAACTTCTCC	qPCR
mexX-RT-R	ATGTCCTTGTCGGCGACAC	
mexY-RT-F	GTGTTGATCGTGTTCCTC	qPCR

mexY-RT-R	CTTGAAGTAGATGTCGTTGG	
czcC-RT-F	GCTACTGATGGGACTCTG	qPCR
czcC-RT-R	ATCGAAGTGCCGTTGAAA	
oprD-RT-F	CTGAACCTAGCCTCCTATG	qPCR
oprD-RT-R	CCGTAGCCGTAGTTCTTAT	
czcR-RT-F	GAAGTCAAGACTGCCGACTAC	qPCR
czcR-RT-R	ACCAGTTCGTAAGGGTGTTG	
oprM-RT-F	TTCGGGTTCCTGGTTGTTC	qPCR
oprM-RT-R	CGTTGATGTCCTTCTGGATCTT	
opmD-RT-F	CAGCGAGCACCGAAGAC	qPCR
opmD-RT-R	GGGCGAAGAAACCGATGAA	
mexR-RT-F	GCCCGACGTCCATGTATT	qPCR
mexR-RT-R	GGGTGATCAGTGCCTTGT	
mexT-RT-F	AAACGAGGAACGCCATGAA	qPCR
mexT-RT-R	GTTCGTGCATCAGGGTCTC	
nalD-RT-F	AAAGGGCGTGTCCCATAC	qPCR
nalD-RT-R	CTGGTTGAGCATCTCGTTGA	
Csy1-F	GGCAAAGGCAGAATGTAAA	qPCR
Csy1-R	GATGGCGAGGTTGTTATG	
Csy2-F	GCCCGTGATAGAGAAACA	qPCR
Csy2-R	CAAAGCAGGTCCGATAAC	
Csy3-F	GGACTTCTATACGCTGCT	qPCR
Csy3-R	TTACTTCTCTTCGGCTTCA	
Csy4-F	ACTTCCGTCTCTTCATCC	qPCR
Csy4-R	CTTTGCTCAACCCGTAAC	
KT2440-rpoA-RT-F	GAACCTGAAAGGTCTCGCTATC	qPCR
KT2440-rpoA-RT-R	CTGAATATCGGCAGCGGTAA	
KT2440-katE-RT-F	GTTGGACCCGACCAAGATAA	qPCR
KT2440-katE-RT-R	CGACCTCGGCAAAGAAATTG	
MG1655-recA-RT-F	GAGAAGATCGGTCAGGGTAAAG	qPCR
MG1655-recA-RT-R	AGAGAAATCCGGCGTTGAG	
MG1655-acrA-RT-F	TCACCAGTGACGGCATTAAG	qPCR
MG1655-acrA-RT-R	ATAGCGCGTAGGGTGATAGA	
PmexA-F	GATAACCGGCCATCGAGCTA	EMSA probe
PmexA-R	GTTCGTTGCATAGCGTTGTCC	
PmexG-F	GAATCCACCTGAAACCCACGCC	EMSA probe
PmexG-R	GAAGCGCTGCATGGGTCGTTC	
PrecA-F	TCCATTGAAGTCCTCGCGAAGT	EMSA probe
PrecA-R	GCAACCGTTCGGAACATTCTTCC	
